# Reviews and Bibliographical Notices

**Published:** 1882-07

**Authors:** 


### REVIEWS AND BIBLIOGRAPHICAL NOTICES.

"THE PHYSICIAN HIMSELF : AND WHAT HE SHOULD ADD TO THE STRICTLY SCIENTIFIC. By D. W. Cathell, M.D., late Professor of Pathology in the College of Physicians and Surgeons of Baltimore ; ex-President of the Medical and Surgical Society ; active member of the Medical and Chirurgical Faculty of Maryland," etc., etc. Baltimore : Cushings & Bailey. 1882. Price $1.25.

Books upon the various scientific divisions of medical knowledge exist in profusion. There is no lack of short manuals, medium-sized text-books, and comprehensive encyclopædias of not only the fundamental branches of medical science and art, but even of the various specialties. Thus, a recent German work on gynecology comprises three large volumes, contributed by ten different authors, while it has lately been stated that a similarly comprehensive work on dermatology is in preparation in this conntry.

The purpose of all this mass of valuable literature is the *acquisition* of medical knowledge. All who write books on practical medicine and surgery content themselves with teaching how to treat diseases. How to attract to one's self patients having ailments to treat, how to avoid infringements of the Code of Ethics in points where that written ethical guide is vague, and how, finally, to secure a proper compensation for the labor performed in the practice of medicine seem altogether beyond, or beneath, the purview of the mass of authors.

Prof. Cathell, the author of the work before us, has most successfully endeavored to fill this vacancy in our medical literature. *How to succeed in the practice of medicine,* would be a discriptive title of this book, were it not that such a catchpenny title fails altogether in doing justice to the character of the work. The text which is the keynote of Dr. Cathell's book is, that professional tact and business sagacity are as much needed for success in the practice of medicine as the highest scientific attainments. It is a sad truth, as stated by the author, that "there are gentlemen in the ranks of our profession who are perfectly acquainted with the scientific aspects of medicine, and can tell you what to do for almost every ailment that afflicts humanity—who, nevertheless, after earnest trial, have never gotten either reputation or practice, because they lack professional tact and business sagacity; and there is nothing more pitiful than to see a worthy physician deficient in these qualities, waiting year after year for practice that never comes."

How to avoid such failure, and how to make success more certain, rapid, and complete, was the author's object in writing and publishing this work. The advice given is highly practical, and open perhaps to the objection on the part of the hypercritical that it is of the character termed "worldly wise," but in no instance does the author deviate from the purest professional honesty in his precepts. There are probably few medical men who cannot profit by the sage maxims contained in the book, but for the young practitioner it must prove invaluable. Some may say of many of the aphorisms that they are trite and commonplace, but we venture to assert that not a few of our readers will find in the following paragraphs, culled at random, some defects of their own alluded to.

"You will be more esteemed by patients who call at your office for any purpose, if they find you engaged in your professional duties and studies, than if reading novels, making toy steamboats, or following other non-professional pursuits; even reading the newspapers, smoking, etc., at times proper for study and business, have an ill effect on public opinion. Public opinion is the creator, the source of all reputation, whether good or bad, and should be respected; for a reputation is a large part of a doctor's capital."

"Accustom yourself to write in a good, clear hand. Write every prescription as though critics were to judge you by it: principal ingredients first, adjunct next, and vehicle last, unless you have some special reason for inverting them. Such a system insures well-balanced prescriptions, disciplines the prescriber, and engenders the respect and favorable criticism of all who notice it."

"Your professional fame is your chief capital: ambition to increase it by all legitimate means is not only fair, but commendable; after you attain it, you will not be apt to lose either it or the practice it insures, so long as you are sober, decent and discreet in conduct, and have the physical health to endure your labors."

"Cheerfulness is also a fountain that is never failing in its influence. Medicine, contrary to the general belief, is not a gloomy, morose profession, but a cheerful one."

"A brusque manner is bad for a doctor, unless sustained by unquestionable skill or reputation. A gentle, urbane but firm manner is suitable to the largest part of the community."

"Never publish weak or trifling medical articles, as whatever one writes is supposed to be a mirror of his own mind. When you write anything for the journals, give your article a proper title, avoid diffuseness, and use no far-fetched quotations from foreign languages. . . . Pity those who are either so ignorant, on the one hand, and those who are so high-flown on the other, that they cannot express themselves in their mother-tongue."

"You should seek proper relaxations and amusements while the age for enjoying them remains ; many doctors foolishly postpone all relaxation from one time to another, intending to take it easy and see pleasure when they get older, and thus forego seeking enjoyments till they lose all taste for them, till they know nothing and are fit for nothing but to work in the doctor's hard, slavish treadmill for life. A little leisure is a great blessing. An occasional day's sport, or a summer trip, or an evening at a convivial gathering, or at the theatre, etc., will act as seasoning to your labors, will break the monotony, afford diversion, and actually make you more philosophical and a better doctor."

We could go on thus and clip from every page nuggets of valuable knowledge gleaned by the author from his own experience and his observation of others ; but enough has been given to show the character of the book, which we can unhesitatingly recommend to all our readers, especially those who are just starting out in practice, or those whose success in their calling does not equal their expectations. We have no doubt the latter class would find the causes of their want of success, and at the same time the means for removing them, pointed out in its pages.

The publishers have done their part in an exceptionally excellent manner. The book is well printed on heavy white paper, and neatly bound.

"A PRACTICAL TREATISE ON DISEASES OF THE SKIN. By Louis A. Duhring, M.D., Professor of Diseases of the Skin in the Hospital of the University of Pennsylvania; Dermatologist to the Philadelphia Hospital; Consulting Physician to the Dispensary for Skin Diseases. Philadelphia; author of 'Atlas of Skin Diseases,'" etc. Third edition. Philadelphia: J. B. Lippincott & Co. 1882. Price, $6.

When the first edition of Prof. Duhring's book was published, a little over five years ago, the present writer took occasion to say in reviewing it that it was the best work of the kind in the English language. In the second and third editions, the latter of which has just come from the press, so many improvements and additions have been made that it may now unhesitatingly be pronounced to be the best text-book on skin diseases in any language. As an encyclopædia of knowledge upon this specialty, Hebra and Kaposi's classical work still holds its pre-eminence, but in clearness of statement, accuracy of pathological details, and fulness in therapeutical resources, the volume under review is excelled by no other work accessible to the student or practitioner. The typographical execution is excellent, and reflects credit upon the publishers.

"A MANUAL OF OBSTETRICS. By A. F. A. King, M.D., Professor of Obstetrics and Diseases of Women and Children in the Medical Department of the Columbian University, Washington, D. C., and in the University of Vermont," etc., etc. With fifty-eight illustrations. Philadelphia: Henry C. Lea's Son & Co. 1882. Pp. 325. Price $2.

This neat little manual aims to supply students with a concise guide to the details of obstetrics. It is based upon the larger works of Playfair, Leishman and Lusk, and very fairly represents the present state of obstetric science, in a highly condensed form. This condensation is, however, not carried beyond the limits of usefulness, for the descriptions in general are exact and easily understood. For use during attendance on lectures, or to rapidly review the facts of midwifery, it is excellently adapted.

The mechanical execution of the work is good, and the price is very reasonable.

